# The effect of vitamin D supplementation on hemoglobin concentration: a systematic review and meta-analysis

**DOI:** 10.1186/s12937-020-0526-3

**Published:** 2020-02-03

**Authors:** Seyed Mostafa Arabi, Golnaz Ranjbar, Leila Sadat Bahrami, Mohammadreza Vafa, Abdolreza Norouzy

**Affiliations:** 1 Metabolic Syndrome Research Center, Department of Nutrition, Faculty of Medicine, Mashhad University of Medical Sciences, Mashhad, 91179481564 Iran; 2 Department of Nutrition, School of Public Health, Iran University of Medical Sciences, Tehran, Iran

**Keywords:** Anemia, Hemoglobin, Vitamin D, Iron status, RCT

## Abstract

**Aims:**

The purpose of this review was to investigate the effect of vitamin D supplements on hemoglobin concentration in subjects aged 17.5–68 years old; using randomized controlled trials (RCTs).

**Methods:**

Relevant RCT studies were identified from January 2000 to January 2019 by using MeSH terms in PubMed, Embase, Cochrane Library, Clinical trials, Scopus databases and gray literature. The studies were reviewed systematically, and quality assessments were evaluated by the guidelines of the Cochrane risk of bias. The effect of vitamin D supplements (*n* = 14) on hemoglobin concentration was considered as primary outcome, while its effects on the levels of ferritin, transferrin saturation and iron status were derived as secondary outcomes. In total, 1385 subjects with age range of 17.5 to 68 years old were examined for 3 h to 6 months; Mean (standard deviation) or median interquartile changes in the hemoglobin concentration in each treatment group was recorded for meta-analysis.

**Results:**

Fourteen RCTs met the inclusion criteria. Current study findings propose that vitamin D supplementation leads to a non-significant reduction in hemoglobin levels in subjects (17.5–68 years old) [std. mean difference (SMD): 0.01; 95% CI: − 0.28, 0.29; *P* = 0.95], also it has no significant effect on ferritin concentrations [std. mean difference (SMD): -0.01; 95% CI: [− 0.20, 0.18; *P* = 0.91]. However, vitamin D supplementation demonstrated positive effects on transferrin saturation [mean difference (MD): 1.54; 95% CI: 0.31, 2.76; *P* = 0.01] and iron status [std. mean difference (SMD): 0.24; 95% CI: − 0.09, 0.39; *P* = 0.002].

**Conclusion:**

Current review concluded that supplementation with vitamin D had no significant effect on hemoglobin and ferritin levels while positive effects on transferrin saturation and iron status were observed. Further clinical studies are required to determine the actual effect of this intervention on hemoglobin levels.

## Introduction

Anemia is one of the most prevalent health problems worldwide. It is believed that this disease is responsible for a large portion of the financial burdens on communities [1, 2]. Iron deficiency is the most common form of the disease and occurs in over 50% of patients worldwide. According to the World Health Organization (WHO), more than one billion people are currently suffering from iron deficiency [1, 3, 4]. Several conditions, such as gastrointestinal disease, chronic heart disease (CHD), chronic kidney disease (CKD), and inflammatory diseases increase the risk of anemia [5–13]. It is believed that these conditions could decrease the quality of life as well as causing great impairments in cognitive functions and increase the prevalence of fatigue and other physical dysfunctions [14–18]. As a result, attempts to improve the prevention and treatment of anemia or the use of potential therapies can help to reduce the burden of this disease [19].

Vitamin D is a fat soluble vitamin that can be acquired from regimen (ergocalciferol from plant sterols) and be synthesized from direct exposure to sunlight (cholecalciferol) [20]. This vitamin is hydroxylated as 25-hydroxyvitamin D (25OHD) in the liver and then converted to its final form, Calcitriol (1, 25 (OH) 2D), in the kidney cells [20, 21]. Vitamin D appears to be associated with the prevention of chronic disease and modulation of immunity, the regulation of cellular growth, and the differentiation and induction of erythropoiesis in bone marrow cells [22–25]. Several observational studies have indicated that there is a reverse relationship between vitamin D levels and anemia in adults [25–28]. Calcitriol (1,25-hydroxyvitamin D) could stimulate erythrocyte precursor cell receptors, which promotes the erythroid progenitor cells maturation and proliferation [29]. It has also been reported that anti-inflammatory effects of vitamin D could down regulate mRNA expression of hepcidin levels [30]. The antimicrobial hepcidin peptides are believed to be associated with absorption and release of iron through suppression and activation of ferroportin (cellular iron exporter) [30]. Therefore, alteration in iron status and prevalence of anemia are expected. According to a previous study, elevated levels of PTH may be related to the risk of developing anemia through reduction in erythropoiesis rate, however it is suggested that vitamin D may increase the production of erythropoietin [29]. A systematic review and meta-analysis of RCTs were carried out in an attempt to summarize the evidence on the effects of vitamin D interventions on iron status (ferritin, Hemoglobin, serum transferrin, transferrin saturation, serum iron and TIBC) and also evaluate the heterogeneity among said RCT results in subjects aged ≥17.5 years old.

## Materials and methods

### Search strategy

This review was conducted in accordance with the guidelines of the Preferred Reporting Items for Systematic Reviews and Meta-Analyses (PRISMA). Notably, PRISMA is primarily used for preparing systematic reviews of such research interventions [31, 32]. The search terms were carried out in the PubMed, Embase, Cochrane Library, and Scopus databases by two independent investigators, and relevant publications cited from January 2000 to January 2019 were identified. The following search terms were used: vitamin D, 25-hydroxy vitamin D, 1, 25-hydroxy vitamin D, ergocalciferol, cholecalciferol, calcitriol, anemia, iron deficiency, hemoglobin, ferritin, transferrin, iron regulation and iron status. Only articles in English and only RCTs that were chronologically limited were considered. The titles and abstracts of the scanned articles were checked, and duplicate citations were then removed. After excluding non-relevant articles, full text of the selected articles (RCTs) were retrieved.

### Study selection

#### Inclusion criteria

A structured approach was taken to set up the research question about this review, using the following five components that are commonly known as the Participants, Interventions, Comparisons, Outcomes, and Study Design Approach (PICOS) [33]:

1. Studies reporting the effects of vitamin D interventions on iron status as primary or secondary outcomes from single or combined vitamin D supplementation with calcium, iron and vitamin K were considered. No restrictions were placed on the gender, age, race, and geographical distribution of the individuals enrolled in the study.
2. Oral vitamin D supplements; such as Cholecalciferol, Ergocalciferol and Calcitriol.
3. Studies carried out in subjects with mean age of ≥17.5 years old.
4. Study design: RCTs.

#### Exclusion criteria

1. Editorials, case reports, letters to the editor, review articles, and animal studies.
2. RCTs without evaluating iron status as their primary or secondary outcomes; or RCT studies with no controls.
3. RCTs that did not report mean (SD) or median interquartile changes in hemoglobin, ferritin levels, transferrin saturation and serum iron levels in each group.

### Data extraction

Two researchers completed the data extraction independently (AM and LM) where qualitative and quantitative information were extracted from each study. Any disagreement between the researchers with respect to the inclusion criteria of a study was resolved by the insight of a third researcher (AN). *P*-value of < 0.05 was considered statistically significant for all of the included RCT studies. The information that were extracted included the name of first author, year of publication, country of origin, study design, sample size, subjects’ age, serum vitamin D baseline levels, dose and types of vitamin D supplements, main study outcomes, and conclusions.

### Quality and risk of bias

The quality of each study was evaluated independently by two researchers (AN and MV) who used bias tool method in the guidelines of Cochrane Collaboration [34]. In accordance with this method, each study was evaluated and good quality was obtained when the total low risk of bias was ≥3 out of 5 items; however, if the total low risk of bias was ≤2 out of 5 items, it was considered as fair quality; and studies with ≤1 low risk of bias out of 5 items were regarded as poor quality [34].

### Statistical analysis

In the current review, all the statistical analysis was performed on RevMan 5.3 software (Cochrane IMS, Oxford, UK) with a random and fixed effect model. Random effect model was used for random variances, when the number of studies entered was limited and there was a difference between the number and characteristics of the individuals [35]. Small and large sample sizes had the same effect in the final conclusion when using this model. The fixed effect model was used for studies with fixed parameters or non-random quantities. We extracted and accumulated continuous data from all RCTs, then analyzed the variables in order to obtain overall weighted mean difference (WMD) with 95% confidence intervals (CIs) by the inverse variance approach. The heterogeneity by Cochrane I^2^ value was calculated with weighted Mantele-Haenszel method as it was suggested in Cochrane handbook [36]. In trials with several duration of supplementations, mean and standard deviations were analysed separately. Publication bias was evaluated according to the Begg and Egger test, by using Comprehensive Meta-Analysis (CMA) V2 software (Biostat, NJ) [37, 38]. It was considered statistically significant if *P* value was less than 0.05.

### Subgroup analysis

Predetermined subgroup analyses were performed according to Deeks et al. [39] study, to evaluate the potential effects of vitamin D interventions on participants with different health conditions. In this method, studies were explored according to the potential heterogeneity of inducer factors, thus separate statistical analyses were performed in each study subgroups. Thereafter, studies were categorized according to the health status of individuals, and then separate meta-analysis was conducted. A significant reduction in the extent of heterogeneity in each subgroup, confirmed the heterogeneity in the health status of individuals. We categorized subjects in RCTs to seven groups as such: healthy adults, anemic patients, chronic kidney disease patients, heart failure patients, hypertensive patients, critically ill patients and athletes. The levels of hemoglobin and ferritin in participants from these groups were assessed and compared when supplemented with vitamin D.

## Results

**Fig. 1:**
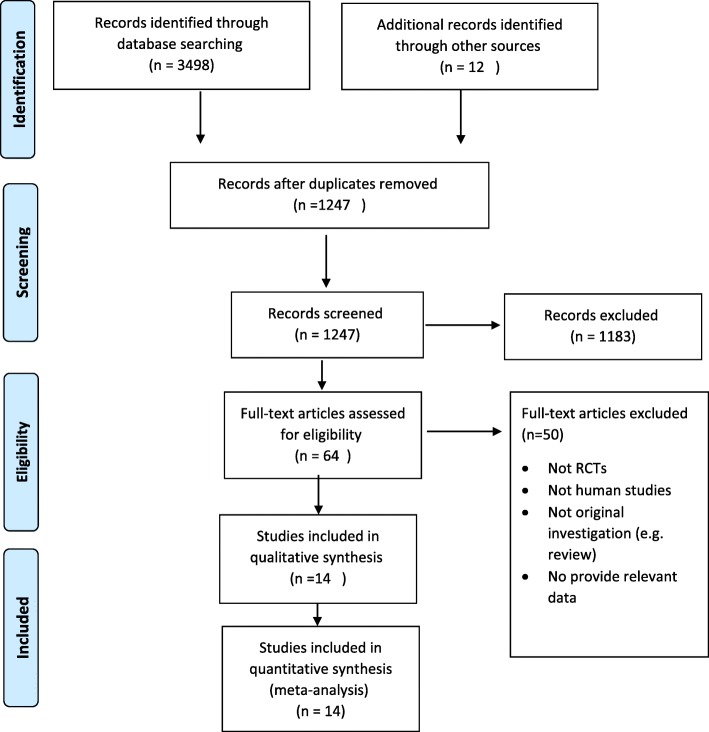
PRISMA flow-diagram of the study selection process

PRISMA flow diagram in Fig. 1, illustrates the selection of included studies and screening process in this review. In total, 3510 articles were found in the initial search, and 3496 of these articles were excluded after reading the titles and abstracts where supplementation of interest was not evaluated. Also, duplicates were removed. Finally, 14 studies met the inclusion criteria (Additional file 1: Table S1) and were suitable for quantitative synthesis [40–53].

### Study characteristics

All studies except two were parallel double blind randomized clinical trials [45, 48]. The main characteristics of the studies are illustrated in Table 1. Studies were published online between 2014 and 2019. The range of sample size was from 10 to 276 participants. Cholecalciferol was the main form of vitamin D that were supplemented in these studies. The duration of supplementation with vitamin D also varied from 3 h to 6 months [40–53].

**Table 1:** Randomized studies comparing the effect of vitamin D supplementation on iron status

Study	Study design	Population	Duration of intervention	Season of intervention	Vitamin D dose and type in intervention group	Comparator group treatment	Baseline 25(OH)D (ng/ml)	Outcome	Conclusion
Trautveffer, 2014, Germany [47].	Parallel RCT - double blind	60 healthy subjects (F=36, M=24), age 20-70 yr.	8 weeks	Winter	Fortified bread with 400 IU, vit D3	Placebo bread	I=20±6.4 P=23.6± 12	↔ Iron ↔ ferritin, ↔ trans%	No effect
Miskulin, 2015, USA [41].	Parallel RCT - double blind	276 CKD Patients, average age 61 yr.	6 months	Not reported	Oral dose of 50,000 IU, vit D2	Placebo pills	I=16.06±5.9 P=16.96±6.4	↔ ferritin	No effect
Toxqui, 2015, Spain [46].	Parallel RCT - double blind	109 anemic women, age 18-35 yr.	8 and 16 weeks	Not reported	Fortified dry milk with 200 IU vit D3 and 15 mg iron	Fortified dry milk with 15 mg iron	Not reported	↓ Iron ↔ ferritin ↔ trans% ↔ Hb	No effect
Sooragonda, 2015, India [45].	Parallel RCT - single blind	30 anemic subjects, age 18-65 yr.	12 weeks	Winter	0.6 Lakh Amp vit D3+ Amp iron	Amp normal saline + Amp iron	I=10.3±5.2 C=11.6±3.7	↔ ferritin ↔ Hb	No effect
Smith, 2016, USA [44].	Parallel RCT - double blind	28 healthy subjects (F=22, M=6), age 18-65 yr.	1 week	Winter	Oral dose of 250,000 IU, vit D3	Oral placebo	I=16.75±1.9 P=16.7±2.03	↔ ferritin	No effect
Smith, 2016, USA [53].	Parallel RCT - double blind	30 mechanically ventilated critically ill adults (F=11, M=19, average age 63.1 yr.	4 weeks	Not reported	Enteral dose of 500,000 IU (I1) and 250,000 IU (I2) vit D3	Enteral placebo	I1=10±90.9 I2=7±77.8 P=8 ±80	↑ Hb	Positive effect
Madar, 2016, Norway [42].	Parallel RCT - double blind	251 healthy subjects, age 18-50 yr.	16 weeks	Late winter	I1=tablet 1000 IU, vit D3 I2= tablet 400 IU, vit D3	Placebo tablet	I1=10.8±6.8 I2=12.4±8.8 P=12.4±8	↔ Iron ↔ Hb ↔ ferritin ↔ trans%	No effect
Hennigar, 2016, USA [40].	Parallel RCT - double blind	152 military trained subjects (F=55, M=98), age 18-42 yr.	9 weeks	Winter	Fortified bar with 1000 IU vit D3 + 2000 mg calcium	Placebo bar	Not reported	↔ Hb ↔ ferritin ↔ trans%	No effect
Ernest, 2016, Germany [50].	Parallel RCT - double blind	200 hypertensive patients (F=94, M=106), average age 60.5 yr.	8 weeks	Summer	Oral dose of 2800 IU IU/ day, vit D3	Placebo tablet	I= 22 ± 5.6 C:20.4±5.6	↔ Hb	No effect
Ernest, 2017, Germany [49].	Parallel RCT - double blind	172 heart failure patients (F=59, M=113), age 18-79 yr.	36 months	Autumn, winter, spring	Oral dose of 4000 IU/ day, vit D3	Placebo drop	I=13.2± 5.6 C:14± 2.4	↔ Hb	No effect
Jastrzebska, 2017, Poland [51].	Parallel RCT - double blind	36 soccer player (M=36), average age 17.5 yr.	8 weeks	Winter	Oral dose of 5000 IU/ day, vit D3	Placebo drop	I=19.6± 3.6 C:19.2±6.4	↔ Hb	No effect
Dahlquist, 2017, Columbia [48].	Cross over RCT - single blind	10 highly-trained cyclists men, age 18-45 yr.	3 hours	Not reported	Sport drink with 5000 IU vit D3 + 1000 mcg vit k2	Placebo drink	I=30± 11.2	↔ Iron ↔ Hb ↔ ferritin	No effect
Walentukiewicz, 2018, Poland [52].	Parallel RCT - double blind	94 elderly women, average age 68 yr.	12 weeks	Not reported	Oral dose of 28,000 IU/ week, vit D3	Placebo drop	I=27.37±8.14 C:24.64±11.61	↑ ferritin	Positive effect
Panwar, 2018, USA [43].	Parallel RCT - double blind	40 CKD Patients, average age 60.5 yr.	6 weeks	Not reported	Oral dose of 0/5 mcg/ day, calcitriol	Placebo tablet	Not reported	↔ Hb ↔ ferritin ↔ trans%	No effect

*Abbreviations*: *RCT* randomized clinical trials, *IDA* iron deficiency anemia, *VDD* vitamin d deficiency, *F* female, *M* male, *IU* international unit, *Vit* vitamin, *Hb* hemoglobin, *Trans %* transferrin saturation, ↑ significant increase, ↔ not significant change, ↓ significant decrease

### Participant characteristics

The average age of participants ranged from 17.5 to 68 years old. Males made up more than 50% of participant’s gender distribution. Mean baseline of 25 (OH) D levels ranged between 10 and 30 ng/ml, as reported in 14 studies (Table 1).

### Intervention characteristics

Different types of vitamin D were used in these studies, four studies received vitamin D fortified food with cholecalciferol [40, 46–48], eight studies received oral vitamin D (cholecalciferol) supplements [42, 44, 45, 49–53] and in one study subjects were supplemented with ergocalciferol and another one with calcitriol [41, 43]. The minimum vitamin D dosage was 20 IU and maximum was 500,000 IU according to these studies (Table 1).

### Outcome measures

All of these 14 RCT studies reported hemoglobin levels, as their primary outcomes. While, iron markers such as levels of ferritin, serum iron, and transferrin saturation were measured as their secondary outcomes. Results are illustrated in Table 1.

### Risk of Bias assessment

**Fig. 2:**
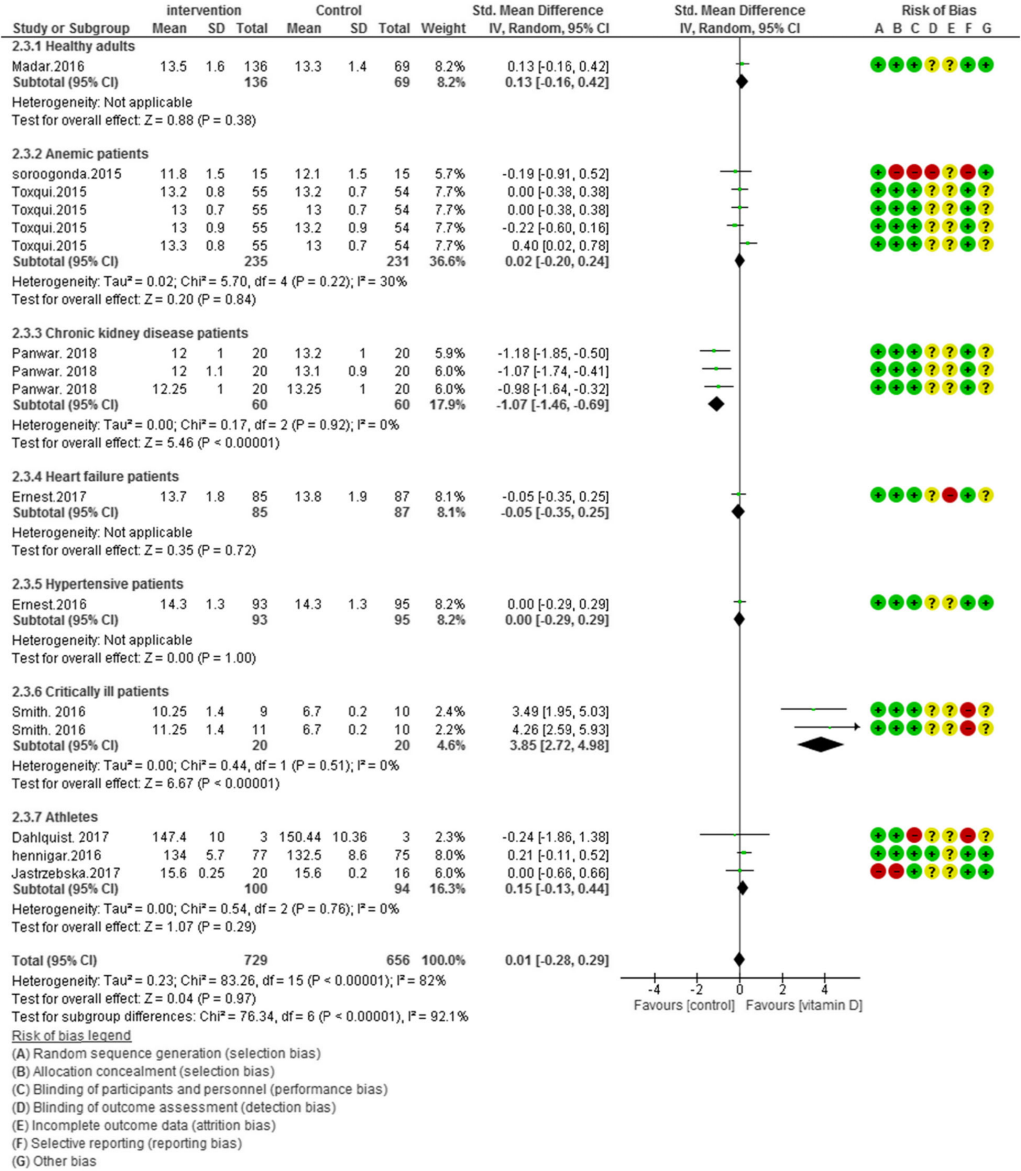
Forest plot showing results of a meta-analysis on the effects of vitamin D supplementation on hemoglobin. Data were reported as SMDs with 95% CIs. (Toxqui study at 4, 8, 12 and 16 weeks after intervention, Panwar study at 1, 4 and 6 weeks after supplementation)

**Fig. 3:**
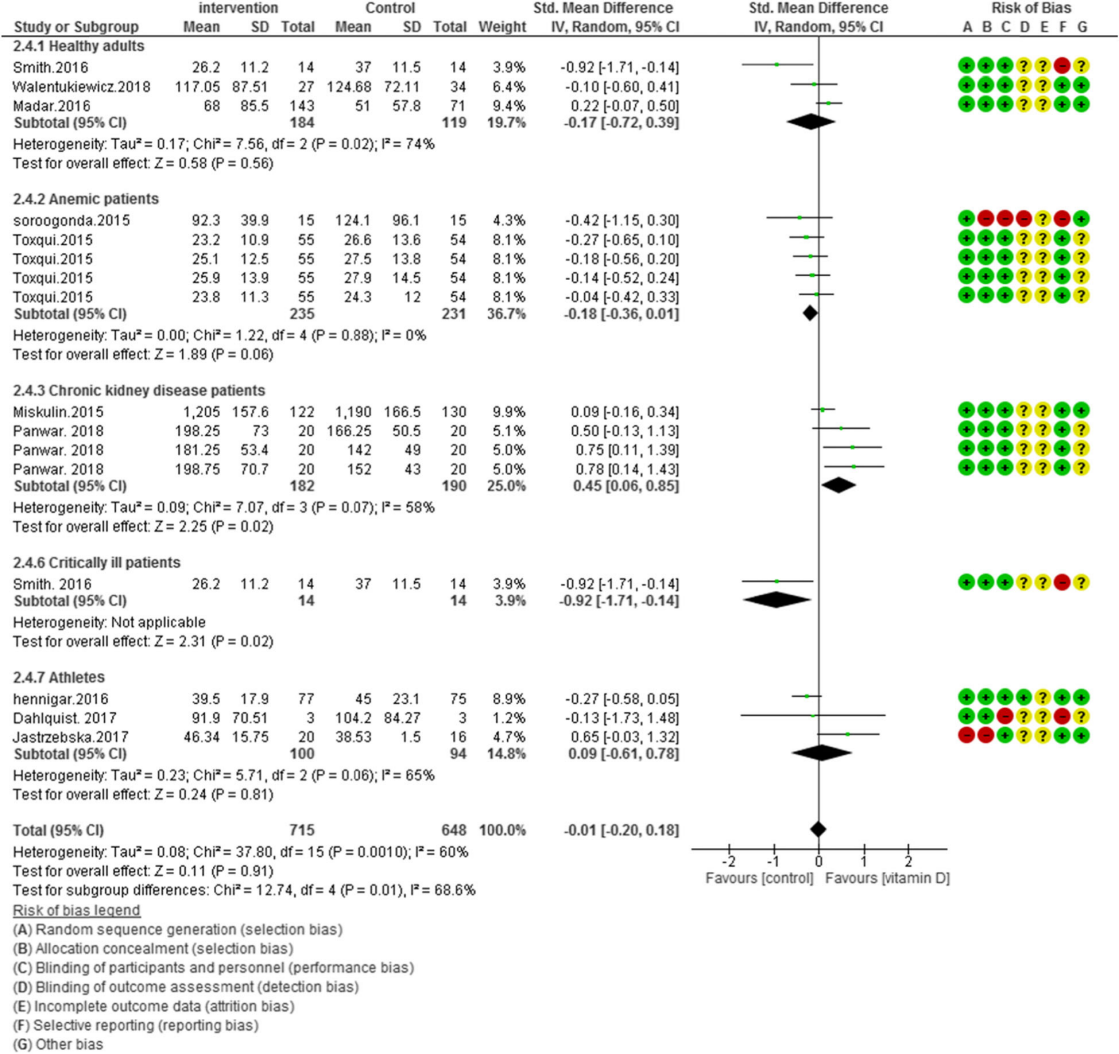
Forest plot showing results of a meta-analysis on the effects of vitamin D supplementation on ferritin. Data were reported as SMDs with 95% CIs. (Toxqui study at 4, 8, 12 and 16 weeks after intervention, Panwar study at 1, 4 and 6 weeks after supplementation)

**Fig. 4:**
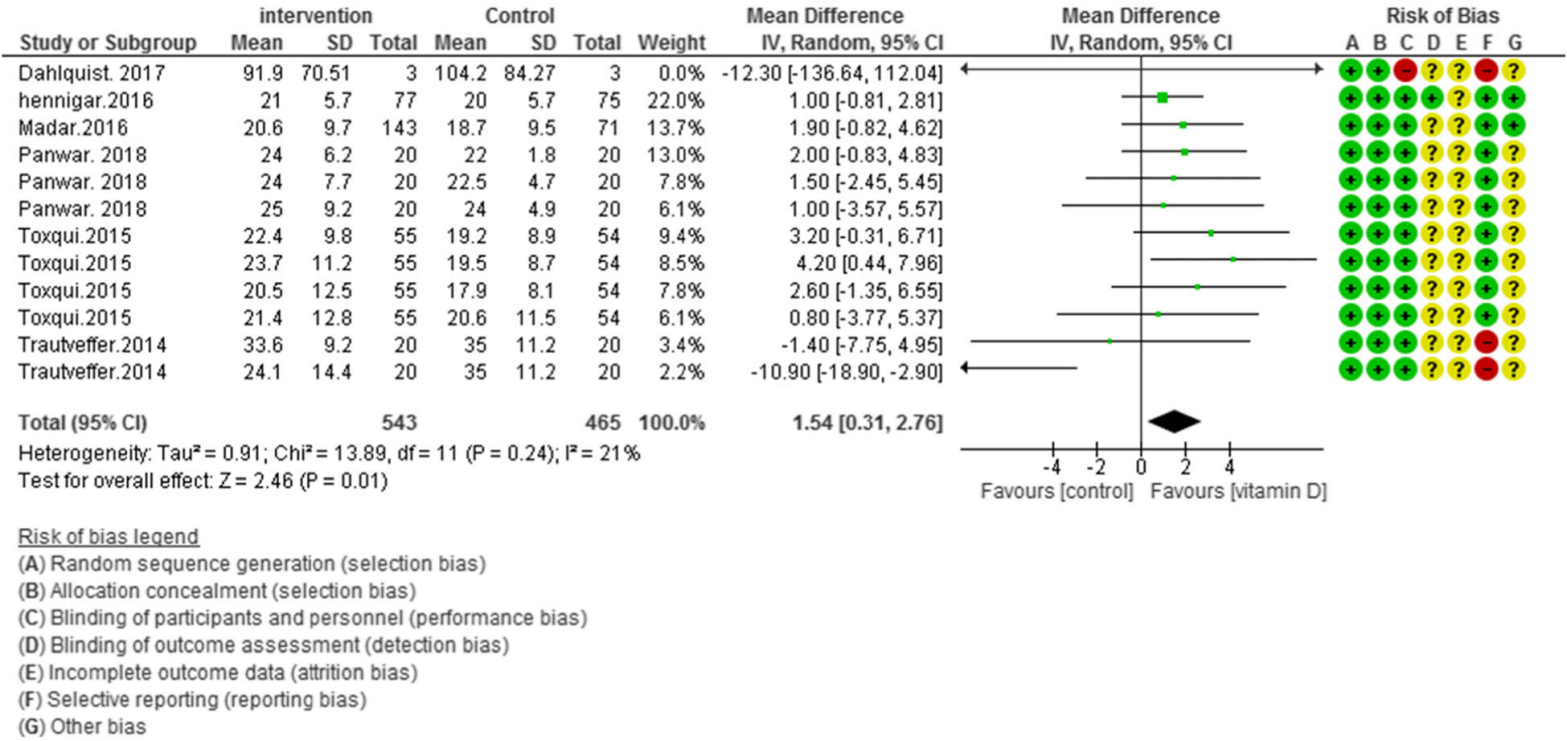
Forest plot showing results of a meta-analysis on the effects of vitamin D supplementation on transferrin saturation. Data were reported as MDs with 95% CIs. (Toxqui study at 4, 8, 12 and 16 weeks after intervention, Panwar study at 1, 4 and 6 weeks after supplementation)

**Fig. 5:**
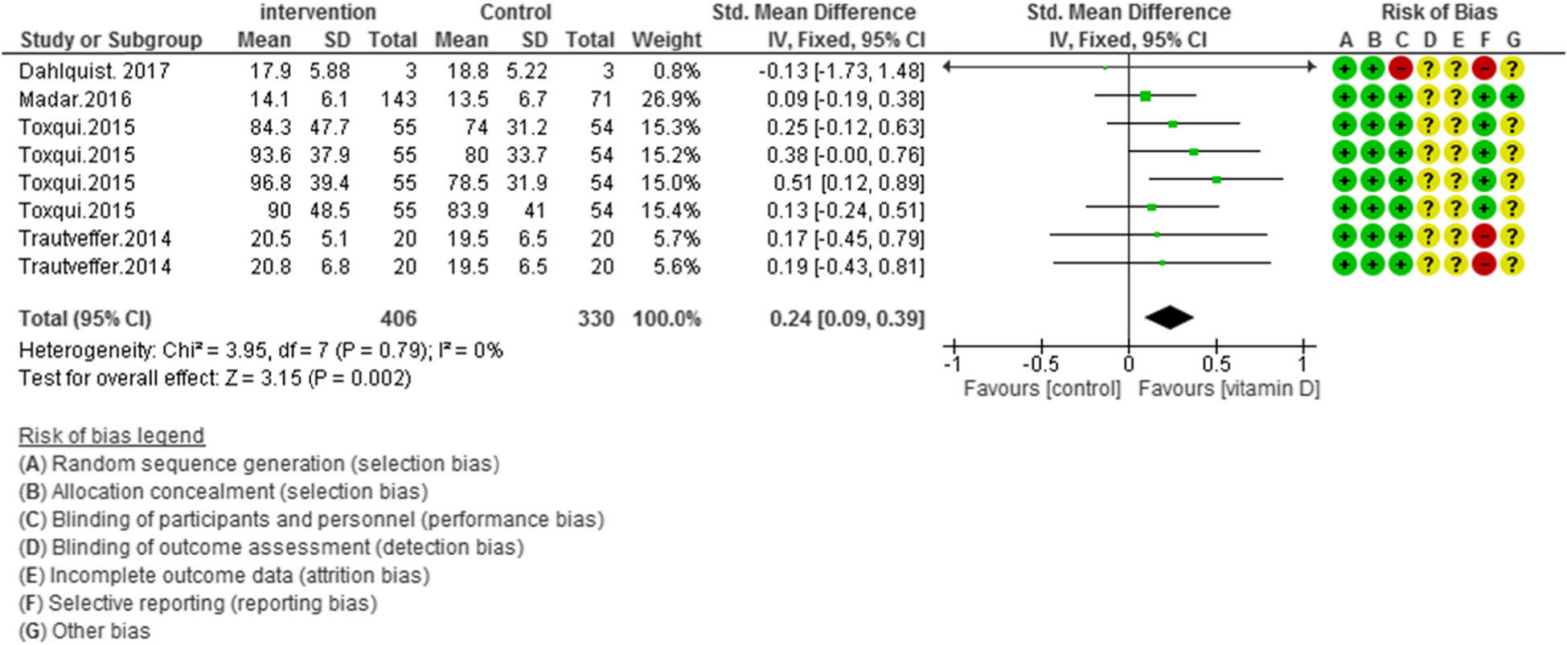
Forest plot showing results of a meta-analysis on the effects of vitamin D supplementation on iron levels. Data were reported as SMDs with 95% CIs. (Toxqui study at 4, 8, 12 and 16 weeks after intervention, Panwar study at 1, 4 and 6 weeks after supplementation)

According to Figs. 2, 3, 4 and 5, the Cochrane risk of bias checklist shows the risk of bias in these randomized clinical trial studies [34]. We evaluated each variable: sequence generation (selection bias), allocating concealment (selection bias), blinding participants and personnel (performance bias), incomplete outcome data (attrition bias), and selective reporting (reporting bias). Each item with low risk and appropriate information was marked as (+), unclear risk and inadequate information marked as (?), high risk and unsuitable information marked as (−). Finally, we assessed the overall quality, nine RCTs had a low risk of bias rate (good) [40–44, 46, 47, 49–53]; two RCTs had a moderate risk of bias (fair) [45, 48].

### Meta-analyses

#### Outcomes

The remaining data from three studies examined differences in iron status markers between intervention and control groups. Markers such as hemoglobin, ferritin, transferrin saturation (TS) and serum iron (SI) levels were included in the pooled analysis component.

#### Primary outcome

##### Overall effect of vitamin D on hemoglobin

Ten clinical trials (*N* = 1385) reported overall hemoglobin levels. When pooled analysis were performed, vitamin D administration did not improve hemoglobin levels [SMD (95% CI) = 0.01 [− 0.28, 0.29]; *p* = 0.76; I^2^ = 82%; Phet < 0.00001]. Significant heterogeneity existed in the data. We categorized our data to seven groups based on participants’ health condition. The subgroup analysis is demonstrated as pooled effect of vitamin D supplementation on hemoglobin in healthy adults 0.13 [95% CI = − 0.16, 0.42]; I^2^ = not applicable, anemic patients 0.02 [95% CI = − 0.20, 0.24]; I^2^ = 30%, chronic kidney disease patients − 1.07 [95% CI = − 1.46, − 0.69]; I^2^ = 0%, heart failure patients − 0.05 [95% CI = − 0.35, 0.25]; I^2^ = not applicable, hypertensive patients 0.00 [95% CI = − 0.29, 0.29]; I^2^ = not applicable, critically ill adults 3.85 [95% CI = 2.72, 4.98]; I^2^ = 0% and athletic subjects 0.15 [95% CI = − 0.13, 0.44]; I^2^ = 0% (Fig. 2).

#### Secondary outcome

##### Overall effect of vitamin D on ferritin

In 11 of included studies (*N* = 1363) vitamin D supplementation did not improve ferritin concentrations in overall [SMD (95% CI) = − 0.01 [− 0.20, 0.18]; *p* = 0.91; I^2^ = 60%; Phet < 0.0010]. Significant heterogeneity existed in the data. For clarifying the impact of vitamin D, subgroup analysis was performed in five groups. The subgroup analysis showed the pooled effect of vitamin D interventions on ferritin levels in healthy adults: -0.17 [95% CI = − 0.72, 0.39]; I^2^ = 74%, anemic patients: -0.18 [95% CI = − 0.36, 0.01]; I^2^ = 0%, chronic kidney disease patients: 0.45 [95% CI = 0.06, 0.85]; I^2^ = 58%, critically ill adults: -0.92 [95% CI = − 1.71, − 0.14]; I^2^ = not applicable and athletic subjects: 0.09 [95% CI = − 0.61, 0.78]; I^2^ = 65% (Fig. 3).

##### Overall effect of vitamin D on transferrin saturation

Pooled data of six studies (*N* = 1008) showed a significant difference between the case and control groups on transferrin saturation, with low heterogeneity [MD (95%CI): 1.54 [0.31, 2.76]; *p* = 0.01; I^2^ = 21%; Phet =0.24] (Fig. 4).

##### Overall effect of vitamin D on serum iron

The weighted results from four studies (*N* = 736) showed, an improving effect of vitamin D on serum iron levels versus placebo group, without any existed heterogeneity [MD (95%CI): 1.54 [0.31, 2.76]; *p* = 0.01; I^2^ = 0%; Phet =0.24] (Fig. 5).

##### Sensitivity analysis and publication bias

Results of sensitivity analysis was conducted by using leave-one-out method, in this method removal of any of the studies from total RCTs or subgroups analysis could cause no substantial change in the impact of vitamin D supplements on hemoglobin and ferritin levels. For instance, two studies with measured hemoglobin and ferritin levels after supplementation with vitamin D and a moderate quality were removed [45, 48]. Thus, the effect of vitamin D intervention did not significantly change the overall outcome of hemoglobin levels: [SMD (95% CI) = 0.03 [− 0.27, 0.33]; *p* = 0.86; I^2^ = 84%; Phet < 0.00001], and ferritin levels: [SMD (95% CI) = 0.01 [− 0.19, 0.21]; *p* = 0.92; I^2^ = 64%; Phet =0.0005]. Despite the weak asymmetry observed in funnel plot, current results showed that vitamin D supplementation effects on hemoglobin concentrations, had no evidence of publication bias (Begg’s test, *P* = 0.58; Egger’s test, *P* = 0.92) [37, 54].

## Discussion

The present systematic and meta-analysis study included 14 studies with 1385 participants from different countries. Vitamin D3 treatments (20 to 500,000 IU) on adults did not have a significant impact on the levels of serum hemoglobin overall. While, pooled analysis revealed a significant effect of vitamin D supplements on transferrin saturation and iron levels. Subgroup analysis according to the health condition of participants, propose that supplementation with vitamin D significantly increased hemoglobin levels in critically ill patients. Sensitivity analysis for studies with low quality indicated an insignificant change on the total effect. To the extent of our knowledge, this is the first pooled analysis which has evaluated the overall effect of vitamin D interventions on iron status in subjects with different health conditions. According to a cross sectional meta-analysis, there was a positive association between vitamin D deficiency and incidence of anemia [55]. This study, was performed on 5183 subjects, indicated participants who were vitamin D deficient had 64% higher risk of developing anemia compared to those who were vitamin D sufficient [55]. In another meta-analysis by Basutkar et al. [56], .vitamin D supplementation in iron insufficient subjects did not improve their clinical outcomes such as hemoglobin and ferritin levels. Similarly, the clinical trials reported in the present systematic review, showed no significant impact of different doses of vitamin D on hemoglobin and ferritin levels, thus, this could be due to the high heterogeneity of the combined studies [40, 42–46, 49–51, 53]. However, it should be noted that active form of vitamin D can affect erythropoiesis through stimulating the erythroid progenitor cells for proliferation and maturation, therefore calcitriol deficiency may damage erythropoiesis, and this may explain positive effect of vitamin D on iron status [57]. Our primary finding is consistent with a recent meta-analysis, where they showed vitamin D treatment had no association with improvements in patients with anemia [45, 46]. However, according to a narrative review conducted by Smith et al. [58], .cholecalciferol supplementation improved anemic condition, through modulating pro and anti-inflammatory cytokines, which leads to reduction in the levels of hepcidin and progress into anemic status [58]. Finally, vitamin D may boost erythropoiesis by increase in iron availability. Some iron inhibitor recycling agents, including the parathyroid hormone (PTH) and fibroblast growth factor 23 (FGF23) may lead to destructive effects on iron metabolism. However, iron recycling effect of vitamin D probably depends on several conditions such as existed inflammation, high levels of parathyroid hormone and fibroblast growth factor 23 [29, 59]. Notably, in healthy subjects due to their balanced levels of parathyroid hormones and Fibroblast growth factor-23 (FGF-23), also no or low inflammation rate, vitamin D could not be effective for improving the iron status. However, according to the subgroup analysis, in critically ill patients with high inflammation rate and high PTH concentrations, vitamin D may improve their iron status through increase in erythropoietin and stimulation of erythroid progenitor formation while, PTH levels are suppressed. In this study, current outcomes suggest that vitamin D interventions significantly induce transferrin saturation and iron levels. Active vitamin D (1, 25(OH) D3) may have beneficial impacts on the levels of parathyroid hormone (PTH), through increase in the absorption rate of calcium and suppression in the release of PTH from parathyroid glands [60]. Previous studies have proposed that depletion of PTH secretion could be related to improved erythropoiesis by inducing erythropoietin and erythroid progenitor formation as well as decreased fibrosis of the bone marrow [29, 61, 62]. Moreover, since fibroblast growth factor 23 is a negative modulator for iron hemostasis and erythropoiesis, vitamin D may reduce this factor thus improve the metabolism of iron, followed by an increase in the levels of iron [63, 64]. The present meta-analysis has several limitations, duration of RCTs were short, also one of the included RCTs was not assessed in the pooled analysis since it lacked placebo group [65]. Different types of vitamin D such as ergocalciferol and calcitriol were supplemented in these trials [41, 43]. The possible reasons for the heterogeneity in data could be associated with the differences in the duration of supplementation, sex, race, geographical location, seasonal change, mean age, sample size, health conditions, vitamin D dosage and co-supplementations. Therefore, we performed the random-effects model, in order to determine the heterogeneity among these studies. This study lacks evidence regarding the mechanism of action between vitamin D and iron levels.

## Conclusion

In conclusion, current systematic review and meta-analysis demonstrated that vitamin D supplements can improve hemoglobin and ferritin status in critically ill and chronic kidney disease patients’ subgroups. Whereas in other subgroups (healthy adults, anemic patients, chronic kidney disease patients, heart failure patients, hypertensive patients, critically ill patients and athletes) non-statistically significant difference were observed on hemoglobin and ferritin levels. The current study suggests that vitamin D supplementation on inflammatory diseases (CKD and critical illness) could effectively improve anemia status. Although there is still limited evidence in order to support and clarify the exact mechanism of action between vitamin D and iron levels. Therefore, further high-quality, well designed and long term RCTs in this field are extensively required.

## Supplementary information

**Additional file 1:Table S1.** Inclusion and exclusion criteria of studies.

## Data Availability

Please contact author for data requests.

## Abbreviations

CHD: Chronic heart disease
CIs: Confidence intervals
CKD: Chronic kidney disease
EPO: Erythropoietin
FGF23: Fibroblast grows factor 23
Hb: Hemoglobin concentration
MeSH: Medical subject headings
PICOS: The Participants, Interventions, Comparisons, Outcomes, and Study Design Approach
PRISMA: Preferred Reporting Items for Systematic Reviews and Meta-Analyses
PTH: Parathyroid hormone
RCTs: Randomized controlled trials
TIBC: Total iron binding capacity
WHO: World Health Organization
WMD: Weighted mean difference
