# Correction to: The effect of vitamin D supplementation on hemoglobin concentration: a systematic review and meta-analysis

**DOI:** 10.1186/s12937-021-00679-4

**Published:** 2021-03-05

**Authors:** Seyed Mostafa Arabi, Golnaz Ranjbar, Leila Sadat Bahrami, Abdolreza Norouzy

**Affiliations:** 1 Department of Basic Medical Sciences, Neyshabur University of Medical Sciences, Neyshabur, Iran; 2 Metabolic Syndrome Research Center, Department of Nutrition, Faculty of Medicine, Mashhad University of Medical Sciences, Mashhad, Iran

**Correction to: Nutr J (2020) 19:11**

**https://doi.org/10.1186/s12937-020-0526-3**

The author would like to correct some misleading sentences, in inclusion criteria number one and two also in table number one, following the original article [1].

Inclusion criteria #1 under the section Study selection, inclusion criteria on page 2 should read “Studies reporting the effects of vitamin D interventions on iron status as primary or secondary outcomes from single or combined vitamin D supplementation with calcium, iron, and vitamin K were considered. No restrictions were placed on the gender, race, and geographical distribution of the individuals enrolled in the study.”

Inclusion criteria #2 should read “All types of vitamin D supplementation”
