# Supplementary material for: The effect of vitamin D supplementation on hemoglobin concentration: a systematic review and meta-analysis
Source: Nutr J. 2020 Feb 3;19:11. doi: 10.1186/s12937-020-0526-3 (PMC6998164; doi:10.1186/s12937-020-0526-3)
Supplement: Supplementary file 1 — Additional file 1:Table S1. Inclusion and exclusion criteria of studies. [file 12937_2020_526_MOESM1_ESM.docx]

**Table S1.** Inclusion and exclusion criteria of studies

| **First author, year (ref)** | **Inclusion criteria** | **Exclusion criteria** |
| --- | --- | --- |
| Trautveffer. 2014, Germany ([45](#_ENREF_45)). | Healthy adults | Pregnant or breastfeeding women, illness. |
| Miskulin. 2015, USA ([39](#_ENREF_39)). | CKD Patients treated with HD, EPO and iron at least 90 days | Patients with hypercalcemia or hyperphosphatemia, pregnancy or breastfeeding, history of hematologic disease or malignancy, any chronic disease except of CKD, kidney transplantation or peritoneal dialysis planned, active infection or blood transfusion within the past 30 days, life expectancy less than 6 months. |
| Toxqui. 2015, Spain ([44](#_ENREF_44)). | Healthy adult women with low iron stores | Pregnant or breastfeeding women, amenorrhea, menopause, iron metabolism-related diseases, chronic gastric diseases, hemorrhagic diseases, renal disease, regularly consumed iron supplements. |
| Sooragonda. 2015, India([43](#_ENREF_43)) | IDA patients | Pregnant women, chronic kidney or liver disease, celiac disease, hemoglobinopathies, presence of infection or inflammation. |
| Smith. 2016, USA ([42](#_ENREF_42)). | Healthy adults | Pregnant or breastfeeding women, granulomatous conditions, a history of kidney or liver disease, diabetes, a history of malignancy, thyrotoxicosis, a history of calcium or bone abnormalities including hyperparathyroidism, osteoporosis, and Paget's disease, an inability to ambulate, an intake of greater than 1000 mg/ day of calcium, and/or used medications. |
| Smith, 2016, USA ([51](#_ENREF_51)). | Mechanically ventilated critically ill adults | Life expectancy lower than 96 hours, receive parenteral nutrition, shock, dialysis patients, HIV, cirrhosis patients. |
| Madar. 2016, Norway ([40](#_ENREF_40)). | Healthy adults | Pregnant or breastfeeding women, regularly using vitamin D containing supplements, subjects treated for vitamin D deficiency, using medication that interfered with vitamin D metabolism, and suffering from any condition such as malabsorption, kidney diseases, cancer, tuberculosis, sarcoidosis, osteoporosis or recent fractures. |
| Hennigar. 2016, USA ([38](#_ENREF_38)). | Healthy trained military adults | Pregnant or breastfeeding women, kidney disease or renal stone or allergy to ingredients of energy bar. |
| Ernest. 2016, Germany ([48](#_ENREF_48)). | Hypertensive vitamin D deficient patients | Pregnant or lactating women, hypercalcemia patients, chronic kidney disease, chemotherapy or radiotherapy treatment, any acute disease, cardiovascular disease, regular intake of vitamin D more than 4 months, change the routine hypertension treatment. |
| Ernest. 2017, Germany ([47](#_ENREF_47)). | Congestive HF vitamin D deficient patients | Patients received EPO or iron supplement. |
| Jastrzebska. 2017, Poland ([49](#_ENREF_49)). | Soccer player subjects | Not mentioned |
| Dahlquist. 2017, Columbia ([46](#_ENREF_46)). | Highly-trained male cyclists | Consuming anti-inflammatory drugs, athletics were not healthy, subjects training lower than 5-6 times a week. |
| Walentukiewicz. 2018, Poland ([50](#_ENREF_50)). | Elderly women | Unstable hypertension (DBP over 100 mmHg), cardiovascular disease, cardiac arrhythmia, and orthopedic diseases. |
| Panwar. 2018, USA ([41](#_ENREF_41)). | CKD Patients | Patients with hypercalcemia or hyperphosphatemia, pregnancy or breastfeeding, routine vitamin D (> 2000 IU) intake more than 3 months, receive iron supplements, severe anemia, receive EPO, receiving hemodialysis or peritoneal therapy, acute kidney injury or renal transplant. |

**Legend: CKD: chronic kidney disease, HD:** hemodialysis**, EPO: erythropoietin, IDA: iron deficiency anemia, HF: heart failure, DBP: diastolic blood pressure.**
